# The Role of Lipoprotein-Associated Phospholipase A₂ in a Murine Model of Experimental Autoimmune Uveoretinitis

**DOI:** 10.1371/journal.pone.0122093

**Published:** 2015-04-15

**Authors:** G. L. Crawford, J. Boldison, D. A. Copland, P. Adamson, D. Gale, M. Brandt, L. B. Nicholson, A. D. Dick

**Affiliations:** 1 Academic unit of Ophthalmology, School of Clinical Sciences, University of Bristol, Bristol, United Kingdom; 2 School of Cellular and Molecular Medicine, University of Bristol, Bristol, United Kingdom; 3 Ophthiris Discovery Performance Unit, GlaxoSmithKline, Stevenage, United Kingdom; 4 Ophthiris Discovery Performance Unit, GlaxoSmithKline, King of Prussia, Pennsylvania, United States of America; 5 Platform Technology Sciences, King of Prussia, Pennsylvania, United States of America; Boston University School of Medicine, UNITED STATES

## Abstract

Macrophage activation is, in part, regulated via hydrolysis of oxidised low density lipoproteins by Lipoprotein-Associated phospholipase A_2_ (Lp-PLA_2_), resulting in increased macrophage migration, pro-inflammatory cytokine release and chemokine expression. In uveitis, tissue damage is mediated as a result of macrophage activation; hence inhibition of Lp-PLA_2_ may limit macrophage activation and protect the tissue. Utilising Lp-PLA_2_ gene-deficient (KO) mice and a pharmacological inhibitor of Lp-PLA_2_ (SB-435495) we aimed to determine the effect of Lp-PLA_2_ suppression in mediating retinal protection in a model of autoimmune retinal inflammation, experimental autoimmune uveoretinitis (EAU). Following immunisation with RBP-3 (IRBP) 1–20 or 161–180 peptides, clinical disease was monitored and severity assessed, infiltrating leukocytes were enumerated by flow cytometry and tissue destruction quantified by histology. Despite ablation of Lp-PLA_2_ enzyme activity in Lp-PLA_2_ KO mice or wild-type mice treated with SB-435495, the number of infiltrating CD45^+^ cells in the retina was equivalent to control EAU animals, and there was no reduction in disease severity. Thus, despite the reported beneficial effects of therapeutic Lp-PLA_2_ depletion in a variety of vascular inflammatory conditions, we were unable to attenuate disease, show delayed disease onset or prevent progression of EAU in Lp-PLA_2_ KO mice. Although EAU exhibits inflammatory vasculopathy there is no overt defect in lipid metabolism and given the lack of effect following Lp-PLA_2_ suppression, these data support the hypothesis that sub-acute autoimmune inflammatory disease progresses independently of Lp-PLA_2_ activity.

## Introduction

Non-anterior uveitis (posterior, pan and intermediate uveitis) is a collective term used to describe a range of intraocular inflammatory disorders affecting the uvea and retina, and whilst more rare than anterior uveitis, is significantly more sight threatening [1]. Uveitis accounts for 10% of the 285 million visually impaired patients worldwide [2] with considerable financial and social implications, as it predominantly effects the working age population [3]. Uveitis is presumed autoimmune or immune mediated when infectious aetiology has been excluded [1, 4–6]. In mice, experimental autoimmune uveoretinitis (EAU) is an antigen-specific Th1/Th17 CD4^+^ T cell-directed murine model, employed to mimic human non-infectious, non-anterior uveitis, which has been utilised extensively to develop a detailed understanding of the immuno-pathogenesis of vascular inflammation, retinal leukocyte infiltration and mechanisms of tissue damage [6–8]. In this model, tissue destruction is dependent upon activation and infiltration of mononuclear cell infiltration [6, 9–12].

Tissue inflammation is held in check and homeostasis maintained, in part through microglia (resident retinal myeloid cell population) but following breakdown of blood ocular barrier [13] circulating macrophages infiltrate the retina early in the course of EAU [7, 14]. The migration of CD4^+^ T cells to the retina causes activation and further accumulation of CD11b^+^ macrophages. Macrophages display broad heterogeneity and their phenotype and behaviour is regulated by a plethora of stimuli found in the local microenvironment. For example, macrophages can inhibit T-cell proliferation in the eye but also produce tissue-damaging superoxides [7, 15, 16]. Notwithstanding, regulating macrophage activation will reduce inflammation [17, 18] and as such, an ability to manipulate macrophage phenotype and/or migration has the potential to abrogate EAU onset.

One means of repressing macrophage activation is through prevention of hydrolysis of oxidised low density lipoproteins (oxLDL), catalysed by the enzyme Lipoprotein Associated Phospholipase A_2_ (Lp-PLA_2_). This reaction produces lysophosphatidylcholine (LPC) and oxidised non-esterified fatty acids (ox-NEFA) two potent pro-inflammatory mediators, which up-regulate expression of chemokines and adhesion molecules, induce macrophage migration and promote pro-inflammatory cytokine release [19, 20]. The phospholipase A_2_ (PLA_2_) family are a group of enzymes involved in catalysing the hydrolysis of fatty acyl moieties from the sn-2 position of oxidized phospholipid molecules [19]. Lp-PLA_2_ is a 45kDa secreted enzyme, expressed by monocytes, macrophages, T cells and mast cells. This enzyme contains a catalytic serine residue [21] and is active in basal physiological conditions. Unlike other PLA_2_ family members, Lp-PLA_2_ functions independently of calcium and is highly specific for substrates with a short chain fatty acyl residue at the sn-2 position, meaning it does not hydrolyse membrane phospholipids [22].

Lp-PLA_2_ suppression has been used to modulate disease in both atherosclerosis and diabetic macular oedema models [23], which in common with uveitis, present with macrophage mediated tissue damage and underlying vascular pathology. It was first noted that Lp-PLA_2_ expression was high in atherosclerotic plaques, particularly those which were necrotic and prone to rupture and a meta-analysis of nearly 80,000 people demonstrated a continuous association between the mass and activity of Lp-PLA_2_ and risk of coronary heart disease [24]. A diabetic and hypercholesterolemic swine model was used to test the efficacy of Lp-PLA_2_ depletion in atherosclerosis. In short, treatment of atherosclerotic pigs with Darapladib, a selective, reversible inhibitor of Lp-PLA_2_ [25] induced a significant reduction in lesion development and necrotic core area [26]. Similar results were also noted in the human Integrated Biomarker and Imaging Study 2 (IBIS-2) [27] whereby ultrasound analysis confirmed that Darapladib treated patients had smaller coronary artery lesions than those receiving a placebo control. Furthermore, Darapladib has been shown to attenuate expression of monocyte chemoattractant protein 1 (MCP-1), VCAM-1 and TNF-α gene expression, [28] and demonstrate reduced cerebral vascular permeability in the diabetic/hypercholesterolemic swine model [23]. These data further implicate Lp-PLA_2_ inhibition as a promising therapeutic target for prevention or resolution of inflammation and vascular permeability.

In light of success in other models of macrophage mediated vascular inflammatory pathologies, albeit not in autoimmune disease, and given that tissue damage in uveitis is dependent upon macrophage infiltration and activation, we utilised Lp-PLA_2_ knockout (KO) mice and a specific Lp-PLA_2_ pharmacological inhibitor SB-435495 (referred to herein as Lp-PLA_2_ *deletion* or *inhibition* respectively) to determine the role of Lp-PLA_2_ in autoimmune retinal inflammation. The prediction being that enzyme depletion would suppress macrophage activation in the face of a T cell driven disease. However, despite the reported beneficial effects of therapeutic Lp-PLA_2_ depletion in a variety of vascular inflammatory conditions, in the murine EAU model, suppression of Lp-PLA_2_ activity was unable to attenuate disease or show delayed disease onset.

## Methods

### Mice

C57BL/6 Lp-PLA_2_ knockout mice (-/-, KO), and heterozygous (-/+, HET) and WT littermate controls were obtained from GlaxoSmithKline (Philadelphia, USA and Stevenage, UK). The Lp-PLA_2_ KO animals were generated by GlaxoSmithKline using 129/BL6 ES cells. Single nucleotide polymorphism (SNP) analysis was used throughout breeding to generate animals that were >98% C57BL/6. The colony was then established in the UK and SNP analysis was re-run to confirm the genetic background of the mice (>99% C57BL/6) [29]. The animals have no physical or immunological phenotype. B10 RIII mice are inbred colonies at University of Bristol Animal Services Unit. All mice were housed in specific pathogen free conditions with continual access to food and water. Female mice aged 6–8 weeks were immunised for EAU, and housed according to Home Office regulations in the University of Bristol animal house facility. Treatment of animals was carried out in accordance with the European Directive 86/609/EEC, the GlaxoSmithKline Policy on the Care, Welfare and Treatment of Animals and conformed to the Association for Research in Vision and Ophthalmology animal policy (ARVO statement for the use of Animals in Ophthalmic and Vision Research). Mice were sacrificed by recognised schedule one methods and all animal work was approved by the University of Bristol ethical approval group.

### Reagents

Human RBP-3_161–180_ (SGIPYIISYLHPGNTILHVD) and RBP-3_1–20_ peptide (GPTHLFQPSLVLDMAKVLLD) were synthesised by Sigma-Aldrich (Poole, U.K) and Severn Biotech (Worcester, U.K) respectively. High performance liquid chromatography was used to confirm peptide purity of >95%, and aliquots of peptide preparations were stored at -80°C. SB-435495 and vehicle solution were provided by GlaxoSmithKline (Stevenage, UK). Complete media comprising Dulbecco’s modified Eagle medium (DMEM) supplemented with 10% heat inactivated foetal calf serum (FCS), 100U/ml penicillin-streptomycin, 2mmol/L L-Glutamine and Sodium pyruvate (Invitrogen, Paisley, U.K).

### EAU induction and treatment

C57BL/6 and B10RIII mice were immunised subcutaneously (s.c) in one flank with 50μg RBP-3 _1–20_ or 50μg RBP-3 _161–180_ respectively. Peptides were prepared in water (2% DMSO), emulsified in CFA (1mg/ml 1:1 v/v) and supplemented with 1.5mg.ml *Mycobacterium tuberculosis* complete H37 Ra (BD Biosciences, Oxford, U.K.). A simultaneous I.P injection of 1.5μg *Bordella pertussis* toxin (Tocris, Bristol, UK) was also administered. Doses of SB-435495, phosphate buffered saline (PBS) or vehicle solution were administered by I.P injection once or twice daily (9am or 9am/pm respectively) as described (S4 Fig).

### EAU clinical assessment

Digital images of the retina were obtained by topical endoscopic fundus imaging (TEFI) using a protocol described previously by Paques *et al* [30] and subsequently validated for EAU [8]. In short, a Nikon D80 digital camera, with a 10-million pixel charge-coupled device image sensor and Nikkor AF 85 /F1.8 D objective and additional +4.00 dioptre magnifying lens, (Nikon, Tokyo, Japan), 5cm long, 3mm diameter teleotoscope (1218AA: Karl Storz, Tuttlington, Germany), and xenon lamp were used to capture colour images of the retinal fundus. Topical application of phenylephrine 2.5% and tropicamide 1% (Minims; Chauvin Pharmaceuticals, Romford,UK), was used to dilate the pupil, while corneal anaesthesia was achieved by topical application of oxybuprocaine 0.4% (Minims). Viscous eye gel (Novartis Pharmaceuticals, Camberley, UK) allowed digital images to be obtained by direct corneal contact with the endoscope. All images were cropped using Photoshop CS software (Adobe, Mountain view, California, USA). Using an adapted clinical grading system, fundal images were scored according to inflammatory changes to the optic disc and retinal vessels in addition to retinal lesions and structural damage [31].

### Sample collection and cell preparation

Blood samples were obtained via cardiac puncture, collected in neonatal KE/1.3 1.3ml EDTA tubes and centrifuged for 8 minutes at 1800rpm and 4°C. The plasma fraction was removed and stored at -80°C for plasma Lp-PLA_2_ enzyme activity and plasma SB-435495 analysis (GlaxoSmithKline). The enucleated right eye was placed in sterile DMEM for retinal dissection and flow cytometric analysis, the left eye was orientated in optimal cutting temperature (OCT) compound and snap frozen in liquid nitrogen for histology. Spleens were also removed for flow cytometry and histology.

Spleen and thymus were mashed and filtered through a 40μm cell strainer to remove cellular debris. Splenocytes were re-suspended in 1ml ACK salt solution (Ammonium-chloride-potassium lysing buffer, 8,024mg/L NH_4_CL, 1,001mg/L KHCO_3_, 3.722mg/L EDTA.Na_2_.2H_2_0) to eliminate erythrocytes, then all samples were washed, re-suspended in 10 ml sterile DMEM and filtered again to remove aggregated cells.

Eyes were enucleated, washed and all extraneous vascular and connective tissue was removed. The retina was then dissected microscopically in HBSS and retinas were physically disaggregated through a 40μM cell strainer and re-suspended in 200μl FACS buffer.

### Flow cytometric analysis

Cells were incubated with 24G2 cell supernatant for 20 minutes at 4°C before incubation with fluorochrome-conjugated monoclonal antibodies (BD Pharmingen, Oxford, UK) against cell surface markers including, CD4 (553051, 1/100), CD8 (553029, 1/100), CD11b (557657, 1/400), Ly6G (551461, 1/100), Ly6C (553104, 1/100) and CD45 (552848, 1/1000) at 4°C for 20 minutes. A streptavidin secondary antibody was added to all samples, (48-4317-82, eBioscience, San Diego, USA, 1/1000) then cell suspensions were acquired using a 3-laser BD LSR-II flow cytometer (BD Cytometry Systems, Oxford, UK). Analysis was performed using FlowJo software (Treestar, San Carlos, California, USA). Cell numbers were calculated by reference to a known cell–standard, as previously reported [14]. In brief, splenocytes at a series of known cell concentrations were acquired using a fixed and stable flow rate for 45 seconds. Based on total cell number acquired during this time, a standard curve was generated and used to interpolate cell concentrations of ocular infiltrating cells acquired at the same flow rate and time.

### Immunohistochemical analysis

12μm sections were cut from eyes snap frozen in OCT medium at -20°C, and mounted onto poly-L-lysine (Sigma, Poole, UK) coated glass slides. Sections were fixed in acetone for 10 minutes and endogenous peroxidases were blocked with 0.3% hydrogen peroxide and methanol for 30 minutes then washed in PBS. Blocking was achieved using normal rabbit serum (Vector Laboratories, Burlingame, California, USA) diluted in 2% bovine serum albumin (BSA) diluted in PBS (PBSA) for 30 minutes, followed by the addition of a rat anti-mouse primary antibody (MCA1388, AbDSerotec, Oxford, UK, 1/250), in 2% PBSA and left to incubate overnight at 4°C. A biotinylated rabbit anti-rat IgG secondary antibody (BA-4001, Vector, UK, 1/200), diluted in 0.1% PBSA was added to all sections for 30 minutes at room temperature then slides were coated with Avidin/Biotinylated enzyme complex (ABC kit, Vector Laboratories, Burlingame, California, USA) for 30 minutes. Sections were washed, coated with DAB reagent (DAKO, Denmark) for 2 minutes, and then immersed in haematoxylin stain. Slides were immediately washed and dehydrated by passage through 75% and 100% ethanol and Histoclear (National Diagnostics, Georgia, USA). Histomount (National Diagnostics, Georgia, USA) mounting media was used to affix coverslips and sections were examined under a light microscope. Clinical scoring was performed blind by an independent assessor based on cellular infiltrate and structural damage to the retina.

### Immunofluorescent staining

Staining for oxLDL was performed using a polyclonal rabbit anti-mouse antibody (ABIN1385598, Antibodies-online, Georgia USA, 1/100). 12-μm sections (as described above) were fixed using 4% PFA, then permeabilised and blocked using PBS supplemented with 0.25% Triton X-100, 1% BSA and 5% normal goat serum (Vector Laboratories, Burlingame, California, USA). Slides were washed in staining buffer (0.1% Triton X-100, 1% BSA in PBS) and incubated overnight at 4°C with 50μl staining buffer containing the oxLDL antibody plus a rat anti-mouse CD31 antibody directly conjugated with FITC (553372, BD Pharmingen, Oxford, UK, 1/100). Negative controls were treated with the latter only.

The following day, slides were washed and incubated for one hour with 50μl staining buffer containing goat anti-rabbit Alexa Fluor 568 (A-11011, Invitrogen, Paisley, UK, 1/200). Sections then received a final wash and were mounted using VECTASHIELD HardSet mounting media with DAPI (Vector Laboratories, Burlingame, California, USA). Immunofluorescence staining was examined using a confocal scanning laser imaging system fitted with krypton-argon lasers (TCS-SP2-AOBS; Leica microsystems, Wetzlar, Germany).

### SB-435495 plasma analysis

All plasma analysis was performed at GlaxoSmithKline. Briefly, SB-435495 was extracted from plasma (25 μL aliquot) using a protein precipitation method. Following vortexing and centrifugation, an aliquot of the supernatant (100μL) was removed and diluted with 50μL of acetonitrile/10mM Ammonium Formate, pH3 (50/50, v/v) and mixed. Samples were quantitated for SB-435495 using a liquid chromatography mass spectrometry (UPLC-MS/MS) method. The LC-MS/MS system consisted of a Waters Acquity UPLC (Milford, MA) and an AB Sciex API 4000 triple quadrupole mass spectrometer (Framingham, MA) operating under Analyst 1.4.2 software (AB Sciex, Framingham, MA). The lower limit of quantification of SB-435495 was 1ng/ml and the upper limit of quantification was 1000ng/ml in the assay.

### Lp-PLA_2_ assay

Quantification of Lp-PLA_2_ enzyme activity in murine plasma was performed at GlaxoSmithKline via isolation of enzyme catalyzed release of [^3^H-] acetate from the substrate 1-O-Hexadecyl-2-O-[^3^H-] acetyl-sn-glycer-3-phophorylcholine ([^3^H-]PAF obtained from Perkin Elmer, Waltham, MA. Product number: NET910250UC). Twenty microliters (20μL) of plasma was used for a single activity measurement using a 1 minute reaction time at 37°C. The Lp-PLA_2_ enzyme reaction was quenched through chloroform:methanol (2:1 ratio) extraction of each individual reaction. Isolated activity (disintegrations per unit time (DPMs)) were extracted into the aqueous layer and converted to nanomoles of product through normalization to a [^3^H-]PAF standard. Radioactivity (DPMs) was counted using a liquid scintillation counter (Beckman, Brea, CA). Inhibition of activity was gauged according to the activity level in the presence of inhibitor (“inhibited”), relative to blank or control plasma.

### Culture of bone marrow derived macrophages (BMDM)

Mice were sacrificed by cervical dislocation; the hind legs excised using aseptic technique, were placed in sterile DMEM and kept on ice. Following removal of the bone heads with a scalpel, bone marrow was flushed with un-supplemented DMEM. The marrow was then filtered through a 70μm cell strainer to remove debris. Cells were cultured in Teflon culture bags (provided by Dr. M. Munder, University of Heidelberg, Heidelberg, Germany) for eight days at 5–10x10^6^ cells per bag in 50ml of complete culture media supplemented with 50ng/ml macrophage colony stimulating factor and 5% Horse serum (Invitrogen).

### Stimulation of bone marrow derived macrophages

Macrophages were seeded at a concentration of 1 x10^6^ cells/500μl per well in a 24 well plate. Cells were treated with either media alone, IL-4 (20ng/ml Peprotech, New Jersey, USA), prostaglandin-E_2_ (PGE_2_) (0.5 μg/ml Sigma-Aldrich, St Louis, USA), LPS (1–100ng/ml Sigma-Aldrich, St Louis, USA) or IFN-γ (0.5–50ng/ml Peprotech, New Jersey, USA) for 2, 4 or 24 hours as specified. Supernatants were removed and stored at -20°C for further assays.

### Nitrite Assay

50μl Griess reagent (Sigma-Aldrich, St Louis, USA) was added to BMDM supernatants (triplicate) and standards (9 point curve with 10,000nm top standard). Plates were read after five minutes, using a Spectramax-190 plate reader (Molecular Devices, California, USA) and Softmax-pro software, with absorbance wavelengths of 630nm and 540nm and a linear standard curve.

### Extracellular Lp-PLA_2_ assay

Supernatants from BMDM samples, stimulated for 24 hours as described previously, were mixed with a 2-Thiol PAF substrate for one minute. In the presence of Lp-PLA_2_ the substrate was hydrolysed, producing a colorimetric change. Enzyme activity was calculated from the change in absorbance values read over a 15 minute period, using a Spectramax-190 plate reader and Softmax-pro software.

### Statistical analysis

Data are displayed using mean ± SEM. Analysis of continuous data with two variables was calculated using a T test. Those with 3 or more variables were calculated using a one or two way ANOVA as appropriate with a secondary Tukey test to determine the differences between treatment groups or Lp-PLA_2_ genotype. Differences between groups were defined as significant at P<0.05.

## Results

**Fig 1:**
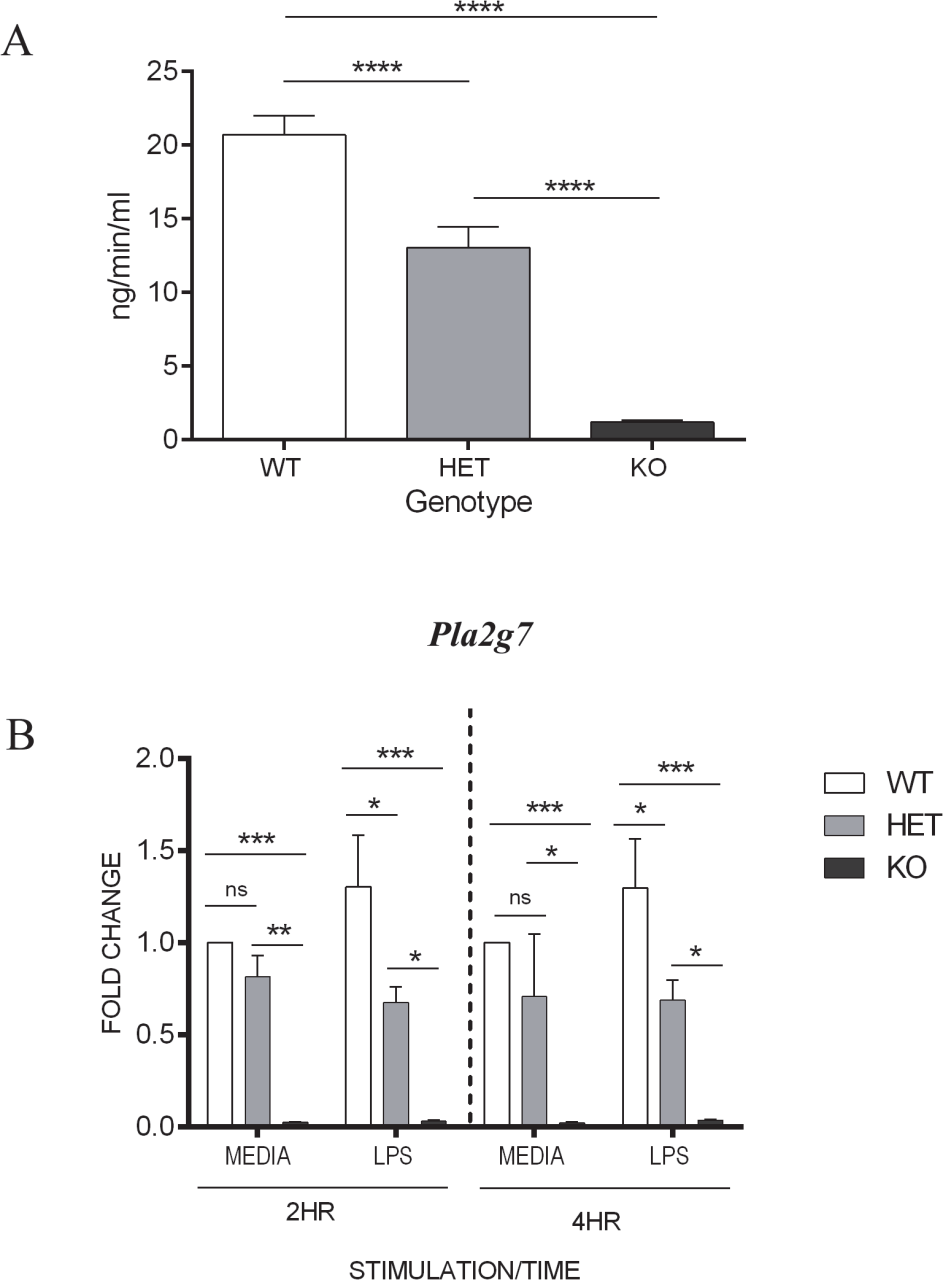
Quantification of Lp-PLA2 enzyme activity and gene expression in murine plasmas and BMDM. **A)** Enzyme analysis of Lp-PLA_2_ activity from murine cardiac blood samples (n = 15–17) **** p<0.001. **B)** RT-qPCR quantification of BMDM *Pla2g7* expression (n = 3). Lp-PLA_2_ WT, HET and KO BMMO were stimulated with LPS or media only for 2 or 4 hours, and gene expression analysed. *Pla2g7* mRNA expression normalized by GAPDH. * p<0.05 ** p<0.01 ***p<0.005

To determine basal enzyme activity status of the Lp-PLA_2_ KO, HET and WT mice, cardiac blood samples were processed for plasma analysis, which confirmed that Lp-PLA_2_ activity was highest in WT animals, reduced in HETs and absent in Lp-PLA_2_ KO mice (Fig 1A). Bone marrow derived macrophages (BMDM), stimulated with LPS (1ng/ml) or media alone for 2 or 4 hours, were used for RT-qPCR quantification of *Pla2g7* gene expression. The results confirmed *Pla2g7* gene expression was absent in Lp-PLA_2_ KO mice, with the highest expression observed in Lp-PLA_2_ WT mice (Fig 1B). To further determine expression and functionality of the secreted enzyme, an *in vitro* extracellular Lp-PLA_2_ activity assay was performed (Cayman chemicals, Michigan, USA). Supernatants from media alone and LPS (1ng/ml) stimulated BMDM secreted detectable quantities of functional Lp-PLA_2_, as indicated by the enzyme induced hydrolysis of a 2-Thio-PAF substrate and subsequent colorimetric change to the supernatant. Furthermore, when BMDM were pre-treated with the SB-435495 inhibitor at a range of doses before stimulation, the enzymatic reaction was absent or reduced (S1 Fig) indicating efficacy of the SB-435495 *in vitro*.

**Fig 2:**
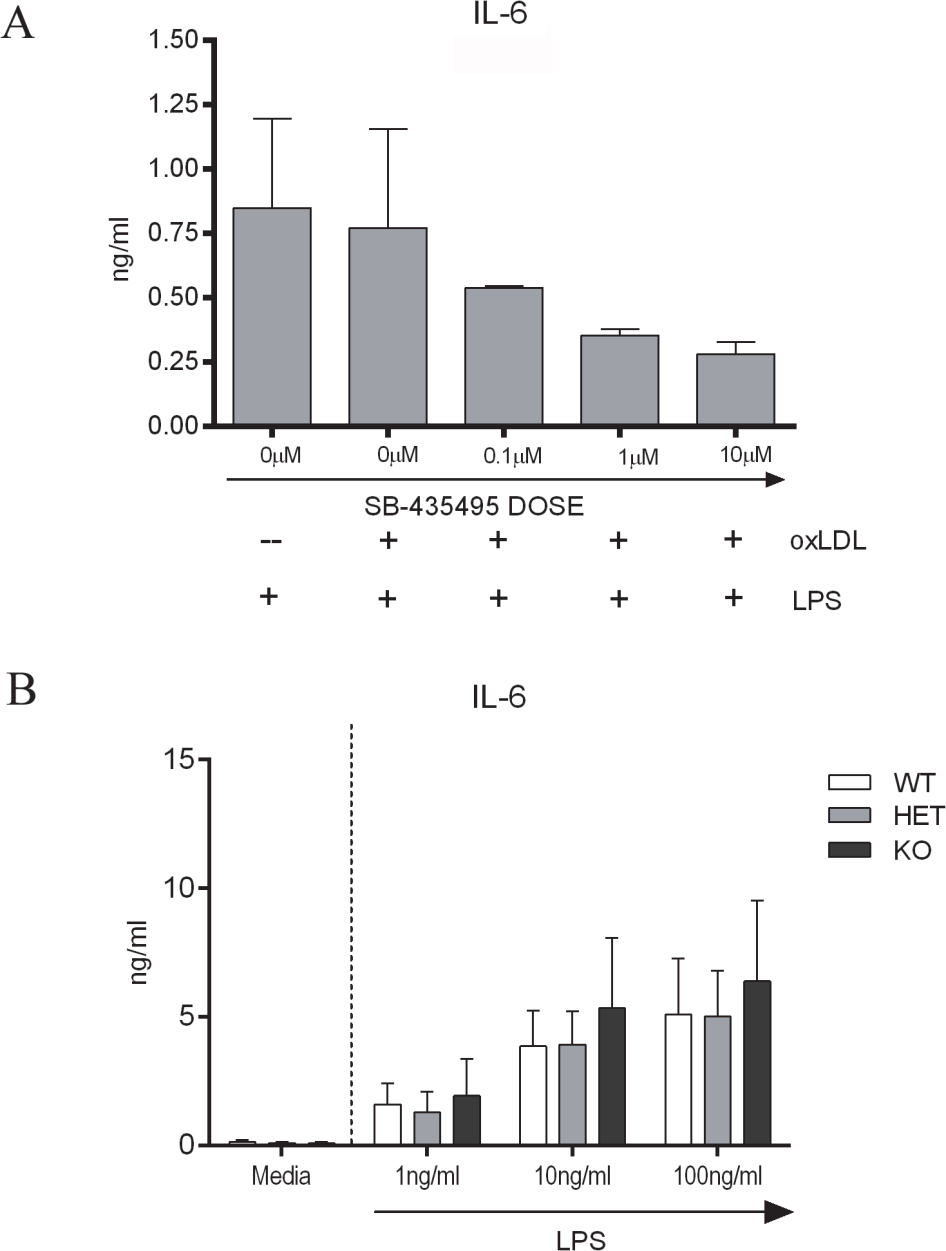
Effect of Lp-PLA2 depletion on macrophage activation and phenotype on BMDM IL-6 protein expression. Supernatants from BMDM cultured for 24 hours with SB-435495 treated oxLDL plus 1ng/ml LPS or media control were used to quantify IL-6 protein expression (n = 3). **A)** SB-435495 was not able to significantly alter IL-6 expression in LPS treated BMDM. IL-6 was undetected in media only controls. BMDM derived from Lp-PLA_2_ WT, HET and KO mice were cultured with a titration of LPS. Supernatants were collected at 24 hours and used to quantify IL-6 protein expression by ELISA. **B)** There was no significant difference in IL-6 expression between Lp-PLA_2_ WT, HET or KO BMDM.

Prior to assessing the *in vivo* therapeutic efficacy of either Lp-PLA_2_ deletion or inhibition, we wished to ascertain whether Lp-PLA_2_ enzyme inhibition altered classical macrophage activation. ELISA estimation of IL-6 and MCP-1 was determined following LPS-stimulation of BMDM treated with a dose range of SB-435495. A trend towards a reduction in IL-6 protein expression was identified in macrophages treated with 10μM SB-435495, when compared to the untreated controls; however the inhibitor failed to significantly alter IL-6 and MCP-1 protein expression, (S2 Fig). To mimic physiological conditions the assay was further developed by spiking with endogenous oxLDL, as Lp-PLA_2_ is transported *in vivo* on oxPL molecules within oxLDL. However the simultaneous addition of SB-435495 and exogenous oxLDL did not significantly alter IL-6 protein expression when compared to controls (Fig 2A). Given our observed sustained IL-6 response, we wished to confirm that IL-6 production was independent of Lp-PLA_2_ and showed that LPS-stimulated BMDM from Lp-PLA_2_ WT, HET and KO mice were equivalent and maintained an IL-6 dose response (Fig 2B).

**Fig 3:**
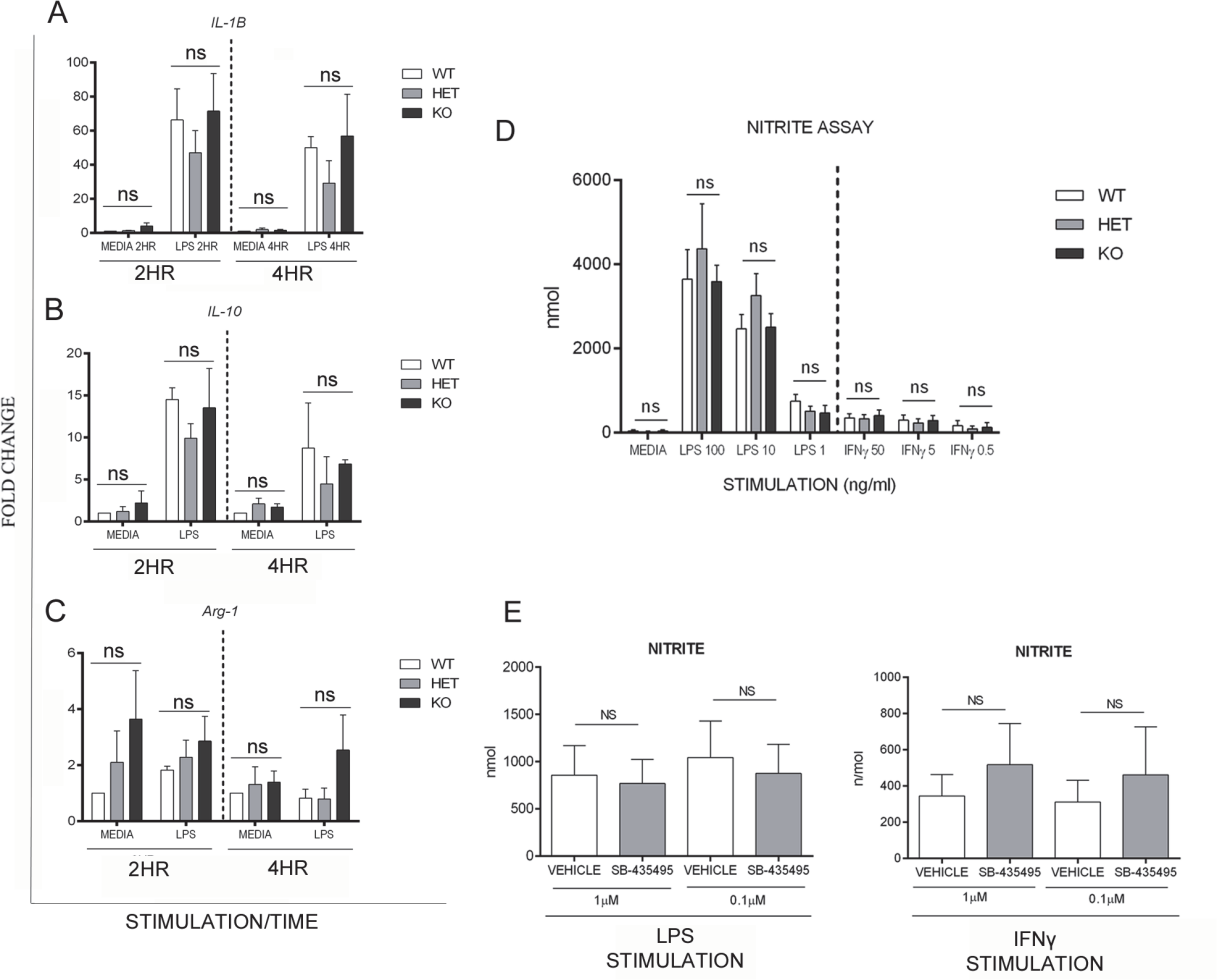
Effect of Lp-PLA2 enzyme ablation on macrophage activation markers as an indicator of macrophage activation status. Expression of IL-1β, IL-10 and Arginase-1 in Lp-PLA_2_ WT, HET and KO mice by RT-qPCR. Data from BMDM, following 2 or 4 hour stimulation with LPS (1ng/ml) or media control **A)** IL-1β (n = 3–4) **B)** IL-10(n = 3) **C)** Arginase-1 (n = 3–4). **D)** A nitrite assay performed as described, using supernatants of BMDM from Lp-PLA_2_ WT, HET and KO mice cultured in presence of LPS/IFNγ for 24 hours, showed no significant difference between genotypes (n = 9 from 5 mice). **E)** Data collected from nitrite assays using supernatants of BMDM treated with either LPS or IFNγ plus SB-435495 (1μM or 0.1μM) showed no significant difference between genotypes (n = 3) ****p<0.001

qPCR analysis was utilised to examine expression of a number of characteristic phenotypic markers of macrophage activation status. Data showed no significant difference in IL-1β and IL-10 gene expression, as a signature of M1 and M2 macrophage activation respectively, in LPS stimulated BMDM from Lp-PLA_2_ WT, HET and KO mice after both 2 and 4 hours in culture (Fig 3A and 3B). Quantification of the expression of Arginase-1 (Arg-1) and Nitric oxide synthase (iNOS or NOS2) was used as a further indicator of macrophage phenotype [32, 33]. Our data showed no significant difference in *Arg-1* expression from Lp-PLA_2_ WT, HET or KO derived BMDM (Fig 3C). Further, a nitrite assay was used to determine whether Lp-PLA_2_ deletion altered NOS2-dependent nitrite production as an indicator of M1 phenotype. Data showed a dose-dependent, LPS mediated nitrite induction, which was independent of Lp-PLA_2_ expression (Fig 3D). Nitrite production was also unchanged in LPS and IFNγ stimulated BMDM from WT mice, cultured with doses of SB-435495 known to give optimal Lp-PLA_2_ inhibition (Fig 3E).

**Fig 4:**
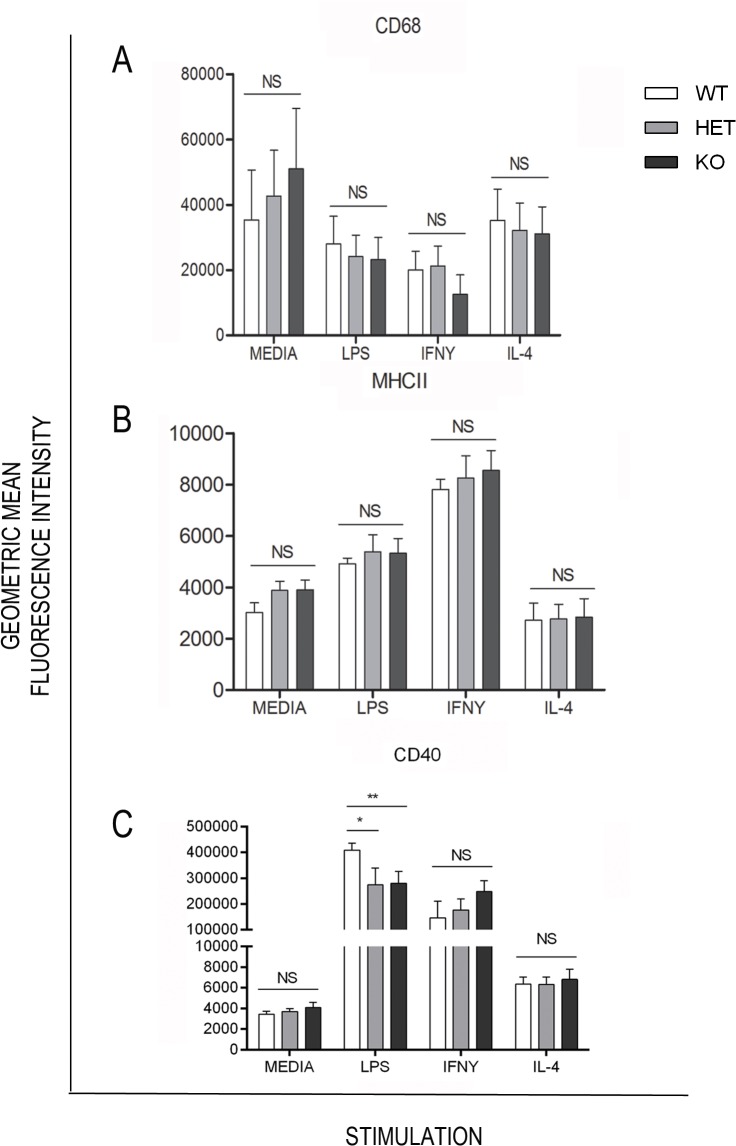
Flow cytometric analysis of BMDM phenotype from cells cultured for 24 hours in the presence of a panel of cytokines. Geometric mean fluorescence intensity values for **A)** intracellular CD68 expression (n = 4), **B)** cell surface MHCII expression (n = 3) and **C)** cell surface CD40 expression (n = 5) * p<0.05 ** p<0.01 ***p<0.005

To further characterise the effect of Lp-PLA_2_ deletion on macrophage phenotype, flow cytometry was used to quantify the expression of macrophage activation markers (CD68^+^ MHCII^+^ and CD40^+^ expression) in BMDM cultured from Lp-PLA_2_ KO, HET and WT mice and stimulated with a panel of cytokines for 24 hours. The expression of MHCII and CD68 was unchanged when Lp-PLA_2_ was deleted_,_ however a significant reduction in CD40 expression was identified in LPS treated macrophages derived from Lp-PLA_2_ HET and KO mice, when compared to WT control cells (Fig 4). Flow cytometric analysis of B and T cell marker expression in the thymus and spleen was also undertaken (CD45, CD3ε, B220, CD4, CD8, CD44, CD62L, CD69, CD25) which showed no significant difference between genotypes in naïve animals (S3 Fig).

Having ascertained the efficacy and functionality of SB-435495 *in vitro* we then wished to determine its effects *in vivo* using the well-established B10 RIII murine model of EAU. Following induction of EAU, mice were randomly allocated into treatment groups and received IP injections of SB-435495 or vehicle control at the doses and time points depicted in S4 Fig. Treatment continued until either day 14 (peak disease) or day 21 (disease resolution) when animals were sacrificed for analysis of retinal cellular infiltrate by flow cytometry and histology. An average Lp-PLA_2_ inhibition of 50.2% and 54.3% (S5A Fig) was measured in plasma samples taken at trough dosing (24h post previous dose) from B10 RIII mice treated once daily with either 20mg/kg or 35mg/kg SB-435495 respectively. Flow cytometry and histology data revealed that treatment of mice with this dose of SB-435495 did not reduce the number of infiltrating cells in the retinas of animals at day 14 or 21 post immunisation when compared to control animals (S5B Fig).

**Fig 5:**
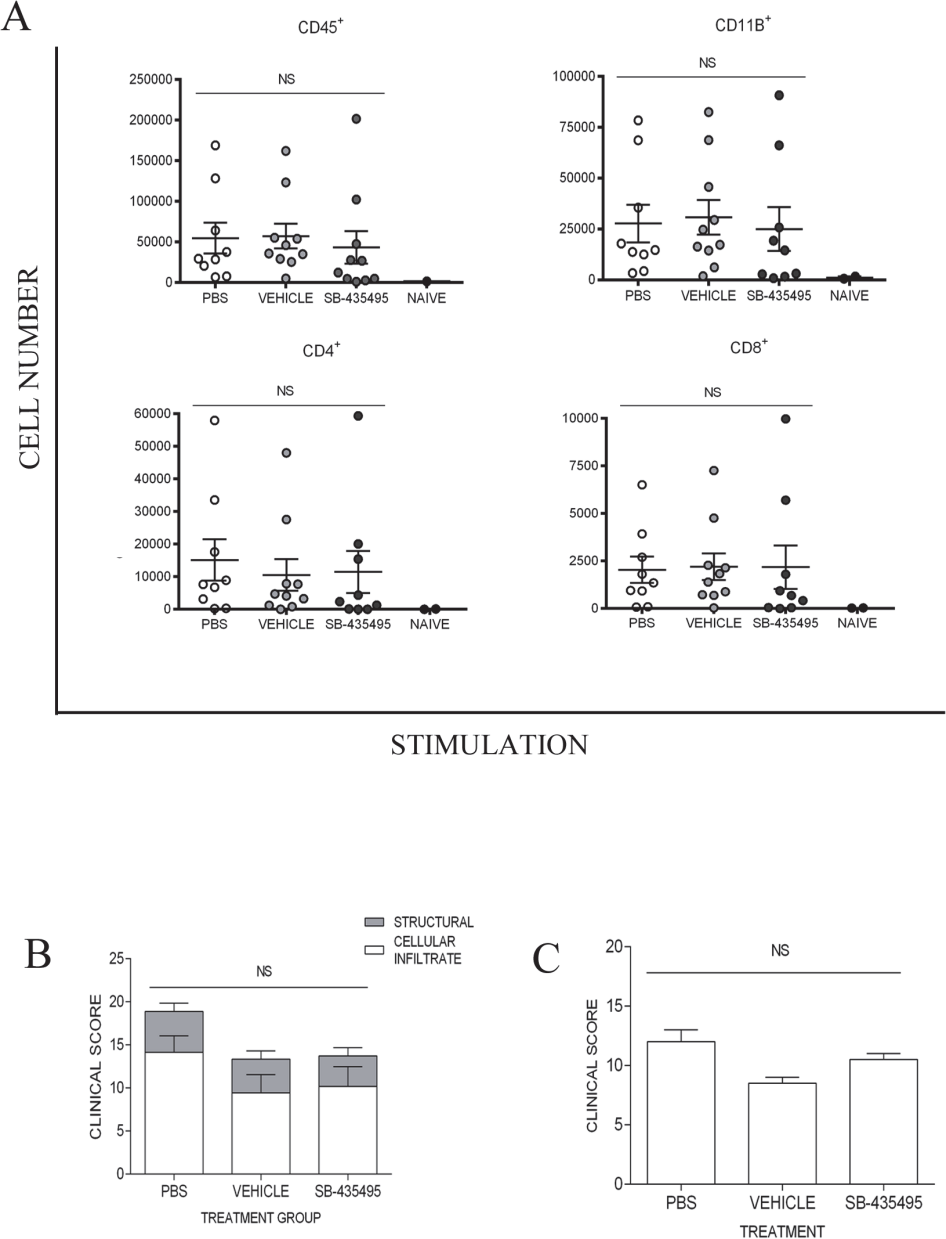
Quantification of retinal cellular infiltrate and onset of clinical disease in a murine model of EAU; effective of SB-435454. **A)** Flow cytometry data showing CD45^+^, CD11b^+^, CD4^+^ and CD8^+^ cellular infiltrate in retinal digests from animals at day 14 post immunisation, treated twice daily with either SB-435495 or controls (n = 9–10 eyes). **B)** Histological staining scores from 12μm ocular sections taken from eyes enucleated at day 14 post immunisation, stained for CD45^+^ (DAB/haematoxylin) (n = 9–10 eyes). **C)** TEFI clinical score, day 13 post immunisation, (average scores provided by two independent assessors from two identical repeat experiments. n = 18–20).

To achieve a more robust inhibition of Lp-PLA_2_ activity, mice were treated twice daily from day 5 post immunisation, with SB-435495 in the hope of maintaining more consistent enzyme inhibition. Mice were given IP injections of 30mg/kg of SB-435495, every 12 hours (S4 Fig). Analysis of plasma samples taken from mice at day 14 post immunisation showed 89.9% inhibition of Lp-PLA_2_ at trough dosing (12 hours post previous dose) (S6A Fig). In spite of this reduction in enzyme activity, there were equivalent numbers of CD45^+^ infiltrating leukocytes in the retina of these mice by flow cytometry (Fig 5A) and histology (Fig 5B). Furthermore, clinical scoring of TEFI images showed no change to longitudinal disease progression and no significant difference in EAU between treatment groups (Fig 5C).

**Fig 6:**
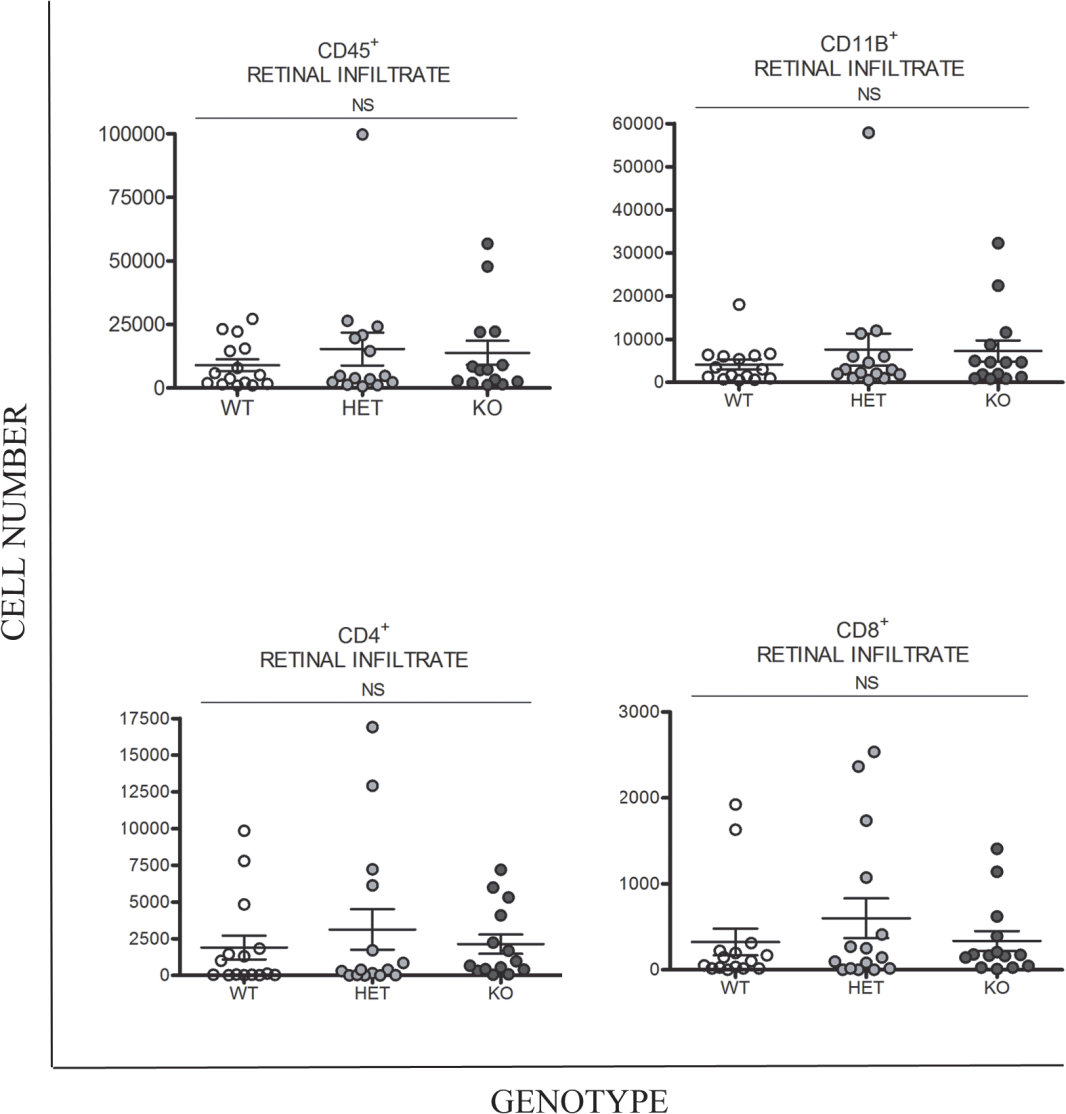
Quantification of retinal cellular infiltrate at day 26 post immunisation in a C57BL/6 murine model of EAU carrying Lp-PLA2 WT, heterozygous and homozygous gene-deletion genotypes. Flow cytometry data showing CD45^+^, CD11b^+^, CD4^+^ and CD8^+^ cellular infiltrate in retinal digests from Lp-PLA_2_ KO, HET, WT animals with EAU at day 26 post immunisation. (n = 14–15 eyes).

As the data suggested partial Lp-PLA_2_ inhibition was insufficient to abrogate retinal leukocyte infiltration, we then wished to assess the effect of permanent and complete deletion of Lp-PLA_2_ in EAU. As such, C57BL/6 Lp-PLA_2_ KO mice were utilised for subsequent experiments, with HET and WT littermates as controls. Mice were sacrificed at day 26 (peak disease) for analysis by flow cytometry and histology. However, in spite of the systemic deletion of Lp-PLA_2_, a comparable number of cells (CD45^+^, CD11b^+^, CD4^+^, CD8^+^) were found to infiltrate the retina during EAU in Lp-PLA_2_ KO mice and HET/WT littermate controls (Fig 6).

**Fig 7:**
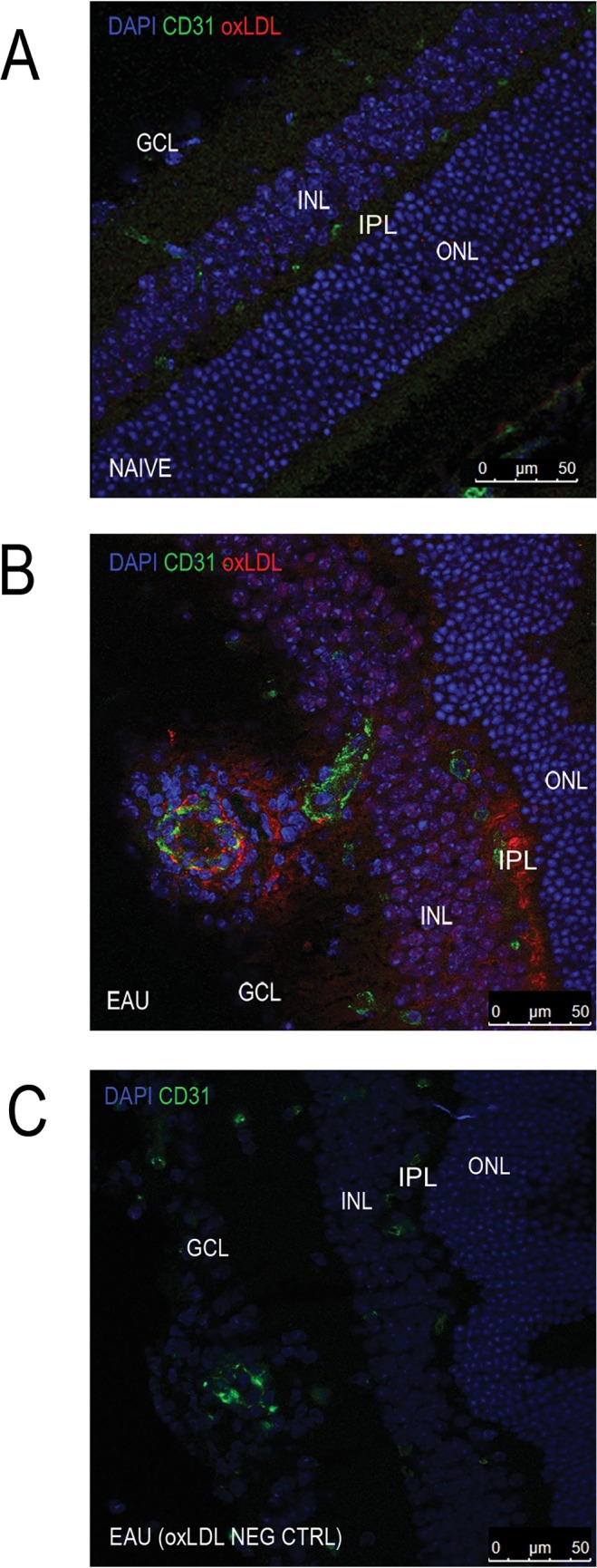
Ocular oxLDL in naive and uveitic mice. Immunofluorescence staining of DAPI (blue) oxLDL (red) and CD31 (green) using 12μm ocular sections from **A)** naïve mice or **B-C)** those immunised for EAU (sequential slides). Ganglion cell layer (GCL), inner nuclear layer (INL), inner plexiform layer (IPL), outer nuclear layer (ONL).

Oxidation of LDL is an important step in the onset and progression of atherosclerosis. Oxidation of the lipoprotein molecule prevents binding to the LDL receptors and infers an increased affinity for macrophage scavenger receptors such as CD36 and LOX-1[34]. This is turn leads to reduced macrophage migration and the formation of lipid-laden foam cells, which are key features of an atherosclerotic plaque. However, less is known about the role of oxLDL in EAU and as such immunohistochemical staining of the Lp-PLA_2_ substrate was used to characterise its availability and distribution in both a healthy and uveitic eye. Data revealed that oxLDL was not detected in the naïve retina (Fig 7A), but in the uveitic retina appeared to co-localise to the vasculature and outer plexiform layer (Fig 7B).

## Discussion

We have successfully shown that SB-435495 is an effective, reversible inhibitor of Lp-PLA_2_ both in BMDM *in vitro* and in an *in vivo* murine model of EAU. A small amount of platelet activating factor acetyl hydrolase (PAF-AH) activity was detected in the serum of Lp-PLA_2_ KO mice (Fig 1A), however this is not thought to be Lp-PLA_2_ as it is not inhibited with SB-435495 at concentrations >20uM which are 200% effective on the isolated enzyme [29]. Significant Lp-PLA_2_ inhibition with SB-435495 was not able to significantly alter the cytokine profile of BMDM *in vitro*, but cells treated with the inhibitor did show a trend towards a dose dependent reduction in the expression of IL-6. Furthermore, *in vivo*, Lp-PLA_2_ deletion or inhibition had no effect on EAU induction or onset.

Lp-PLA_2_ is so called because in humans 80% of the circulating enzyme is carried on apolipoprotein-B100 moieties on low density lipoprotein (LDL) molecules [35]. The remaining 20% is carried on high density lipoprotein (HDL) and very low density lipoprotein (VLDL) molecules, while in mice the ratios are reversed, due to minimal expression of LDL in this species. Increased Lp-PLA_2_ mass and activity has been identified as a biomarker for coronary heart disease, and is found in patients with high cardiovascular risk factors [24]. It is also known to be associated with major adverse events in patients with coronary artery disease in a CRP independent manner [35]. Lp-PLA_2_ is considered a key enzyme in vascular inflammation, due to its ability to catalyse the hydrolysis of oxPL on oxLDL molecules in the intima of blood vessels resulting in the production of ox-NEFA and LPC, which are cytotoxic and have a potent effect on many different cell types.

As such, the notion of Lp-PLA_2_ suppression as a therapeutic target has received much interest in recent years. A porcine model, using streptozotocin treatment and a high fat, high cholesterol diet was used to induce diabetes and accelerate subsequent onset of atherosclerosis. Treatment with the Lp-PLA_2_ inhibitor, Darapladib, resulted in a reduction of Lp-PLA_2_ activity and atherosclerotic plaque size, IL-6 expression and systemic hCRP. Whether such response would be evident in a T cell driven autoimmune model, where both macrophage activation and inflammatory vasculopathy are evident, was the rationale for the current work.

oxLDL molecules have been shown not only to carry the enzyme, but also to up-regulate Lp-PLA_2_ expression in the human THP-1 monocyte cell line [36] and data show that when oxLDL is treated with SB-435495, Lp-PLA_2_ activity is reduced [36]. Furthermore, macrophage expression of Lp-PLA_2_ is increased by TLR4 ligation with LPS, due to a P38 MAPK mediated transcriptional up-regulation [37], suggesting that the enzyme is likely to be highly expressed during inflammation. As such, we hypothesised that by suppressing Lp-PLA_2_ enzyme activity it may be possible to alter macrophage phenotype and subvert the subsequent macrophage-mediated tissue damage that occurs in EAU. To this end, both LPS and oxLDL were used to stimulate BMDM, and we did note a trend towards a dose response of SB-435495 in IL-6 expression (Fig 2A).

MCP-1 expression plays an important role in myeloid cell migration in uveitis and the products of oxLDL breakdown, LPC and NEFA, up-regulate macrophage MCP-1 production, a response we hoped to attenuate by depleting Lp-PLA_2_. However, SB-435495 failed to reduce MCP-1 expression from LPS stimulated BMDM (S2B Fig). oxLDL itself is also capable of inducing MCP-1 expression in macrophages [38] and we therefore postulate the possibility that an equilibrium exists between the influence of oxLDL and the associated hydrolytic bi-products on myeloid cells *in vitro*. Lp-PLA_2_ depletion may cause oxLDL to accumulate, with the potential to induce MCP-1 production, hence negating any beneficial effects of SB-43545. oxLDL also induces myeloid IL-1β production [39] and hence could explain why Lp-PLA_2_ HET BMDM, with intermediate enzyme activity, show the lowest gene expression of *IL-1β* see by qPCR (Fig 3A). In addition to the effect on MCP-1 and IL-1β expression, recent publications have identified a link between oxLDL, and differentiation of cells of the CD4+ TH17 lineage. Using an *in vitro* co-culture of T cells and dendritic cells treated with TGF-β and oxLDL, an up-regulation of TH17 cell differentiation has been identified, which was absent in samples treated with native LDL or an oxLDL depletion antibody [40]. As such, it is possible that both the substrate and bi-products of the Lp-PLA_2_ reaction have the potential to exacerbate autoimmune disease.

Our data show no significant change to *in vitro Arg-1* expression in response to altered Lp-PLA_2_ activity (Fig 3C) and similarly, BMDM expression of *IL-10*, an anti-inflammatory cytokine, was unchanged in KO mice when compared to WT control cells (Fig 3B). Furthermore, in BMDM derived from Lp-PLA_2_ WT, HET and KO mice, there was a dose-dependent LPS mediated nitrite production, but this remained independent of Lp-PLA_2_ activity (Fig 3D). As such, these data infer that depletion of the Lp-PLA_2_ enzyme does not significantly alter macrophage phenotype.

The activity of Lp-PLA_2_ is regulated to an extent by the degree of HDL and LDL binding, which delivers either anti or pro inflammatory/atherosclerotic behaviour respectively as observed in man. Unlike LDL, HDL acts to remove cholesterol from lipid laden foam cells in the vessel intima and acts as an anti-oxidant, reducing the ability of LDL to become oxidised by removal of lipid hydroperoxidases [41]. The behaviour and phenotype of HDL and LDL carrier molecules can be altered in chronic conditions such as type II diabetes, where increased small dense LDL (sdLDL) molecules afford greater affinity for Lp-PLA_2_ [42]. Similarly, diabetic patients have been shown to have HDL molecules with reduced anti-oxidant behaviour owing to an imbalance of lipid/protein and apoA-I/apoA-II ratios [42]. These lipid modifications contribute to the onset of atherosclerosis, but are unlikely to be observed in the context of sub-acute T cell driven tissue inflammation such as that seen uveitis.

Whilst EAU is comparable in many respects to both atherosclerosis and diabetic macular oedema, demonstrating macrophage and TH17^+^ cell mediated vascular pathology, EAU does not share the same underlying dyslipidaemic traits and therefore it is possible this is why we did not observe an effect in EAU despite the involvement of inflammation, macrophage infiltration and vessel inflammation in this model.

In the normal retina there appears to be limited expression of oxLDL (Fig 7A), which increases when disease is fulminant (Fig 7B). We had predicted that activation, phenotype and migration of circulating myeloid cells might be altered by systemic Lp-PLA_2_ depletion and hence the ocular availability of the substrate would be unimportant. However quantification of retinal leukocyte infiltration during peak disease, by both flow cytometry and histology revealed no difference between Lp-PLA_2_ depleted samples and controls, indicating that the control of migration of cells into the tissue, driven by antigen specific T cell activation, was independent of Lp-PLA_2_ activity.

Interestingly, and in accordance with our work, recent publications have stated that in spite of promising preliminary findings, Darapladib treatment and associated Lp-PLA_2_ reduction failed to significantly reduce the incidence of myocardial infarction, stroke and cardiovascular death in a cohort of patients with coronary heart disease, when compared to a placebo treated control group following an extensive randomised, multicentre phase III clinical trial, [43]. Similarly, whilst we were able to show a clear reduction in systemic Lp-PLA_2_, which resulted in some alteration to macrophage cytokine profiles, our data show that manipulation of Lp-PLA_2_ does not alter the onset or progression of EAU. It seems therefore that the acute inflammatory pathways involved in EAU onset proceed independently of Lp-PLA_2_ mediated mechanisms.

## Supporting Information

S1 Fig Expression and functionality of secreted Lp-PLA_2_. Supernatants from **A)** media alone and **B)** LPS (1ng/ml) stimulated BMDM were found to secrete detectable quantities of functional Lp-PLA_2_, as indicated by a colorimetric change to the supernatant as a result of hydrolysis of a labelled 2-Thio PAF substrate. This response was abrogated in the presence of SB-435495 treatment.
(TIF)

S2 Fig Effect of Lp-PLA_2_ depletion on macrophage activation and phenotype. Supernatants from BMDM pre-treated with a dose titration of SB-435495 plus 1ng/ml LPS were used to quantify expression of IL-6 and MCP-1 by ELISA. n = 3 p>0.05. SB-435495 did not significantly reduce either **A)** IL-6 or **B)** MCP-1 protein expression from LPS treated BMDM (n = 3)
(TIF)

S3 Fig Immune phenotype in naïve Lp-PLA_2_ WT, HET and KO mice. Quantification of B and T cell populations from **A)** the thymus and **B)** the spleen using geometric mean fluorescence intensity (GMFI). GMFI quantification of CD4^+^ T cell activation markers from **C)** thymocytes and **D)** splenocytes and CD8^+^ T cell activation markers from **E)** thymocytes and **F)** splenocytes.
(TIF)

S4 Fig Dosing regimen for *in vivo* EAU experiments using SB-435495. Mice were immunised and randomly allocated to a treatment group. Each mouse was given a standardised daily or twice daily volume of SB-435495 by i.p injection and the dose calculated based on the weight of the animal.
(TIF)

S5 Fig Daily treatment with 20mg/kg or 35mg/kg SB-435495 did not significantly reduce the number of cells infiltrating the retina. **A)** Percent inhibition calculated from murine plasma, taken at time of sacrifice (n = 3) **B)** Quantification of cells infiltrating the retina at day 14 and 21 post immunisation (n = 3) ** p<0.01
(TIF)

S6 Fig *Twice daily* treatment with 30mg/kg SB-435495 significantly reduced Lp-PLA_2_ activity in murine plasma. **A)** % Lp-PLA_2_ inhibition and **B)** Lp-PLA_2_ enzyme activity calculated from murine plasma, taken at time of sacrifice (n = 14–15). *** p<0.005 **** p<0.001
(TIF)
