# Supplementary figures and images for: The Role of Lipoprotein-Associated Phospholipase A₂ in a Murine Model of Experimental Autoimmune Uveoretinitis
Source: PLoS One. 2015 Apr 15;10(4):e0122093. doi: 10.1371/journal.pone.0122093 (PMC4398387; doi:10.1371/journal.pone.0122093)

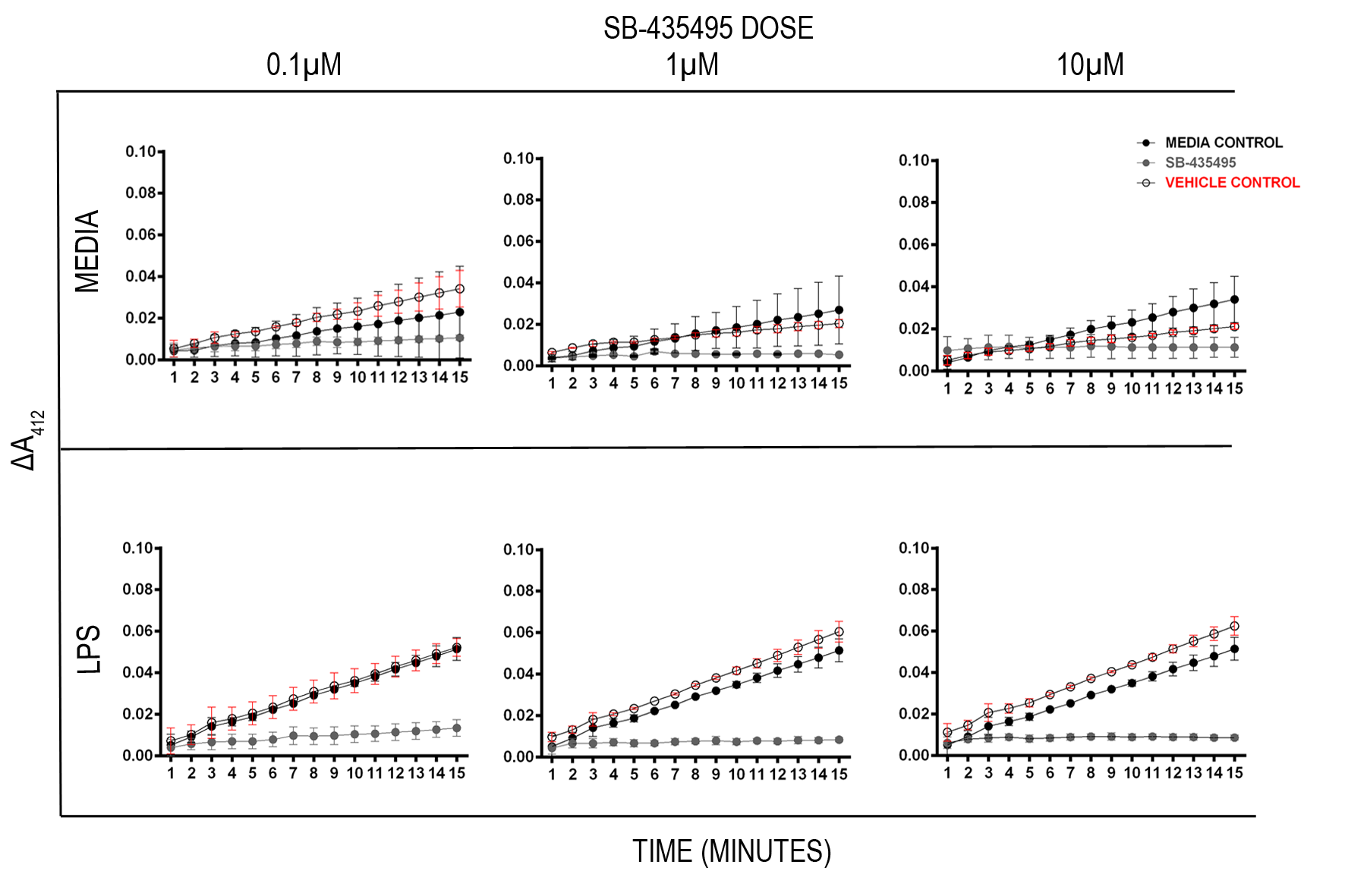

Supplement: S1 Fig — Supernatants from A) media alone and B) LPS (1ng/ml) stimulated BMDM were found to secrete detectable quantities of functional Lp-PLA2, as indicated by a colorimetric change to the supernatant as a result of hydrolysis of a labelled 2-Thio PAF substrate. This response was abrogated in the presence of SB-435495 treatment. (TIF) [file pone.0122093.s001.tif]

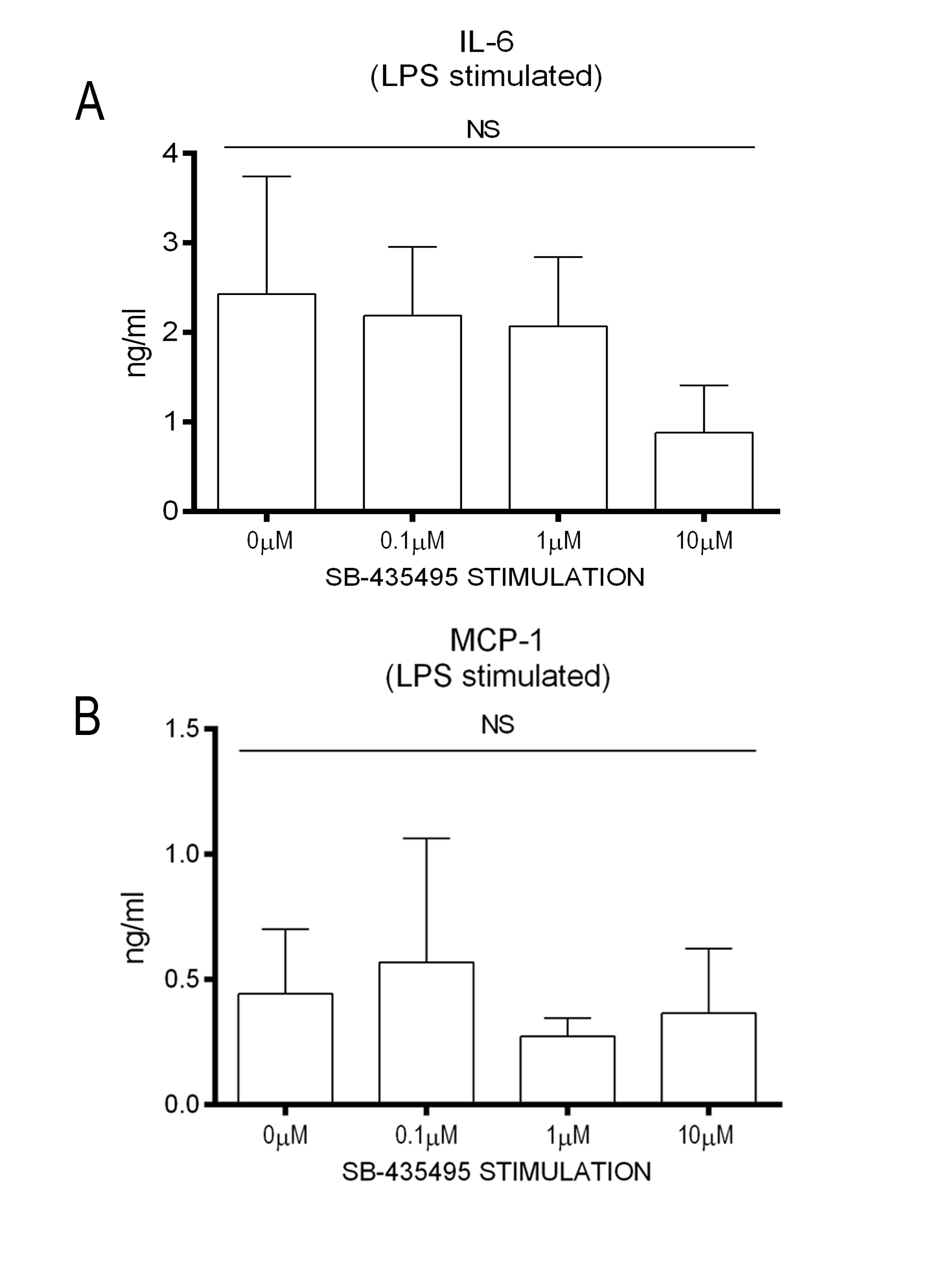

Supplement: S2 Fig — Supernatants from BMDM pre-treated with a dose titration of SB-435495 plus 1ng/ml LPS were used to quantify expression of IL-6 and MCP-1 by ELISA. n = 3 p>0.05. SB-435495 did not significantly reduce either A) IL-6 or B) MCP-1 protein expression from LPS treated BMDM (n = 3) (TIF) [file pone.0122093.s002.tif]

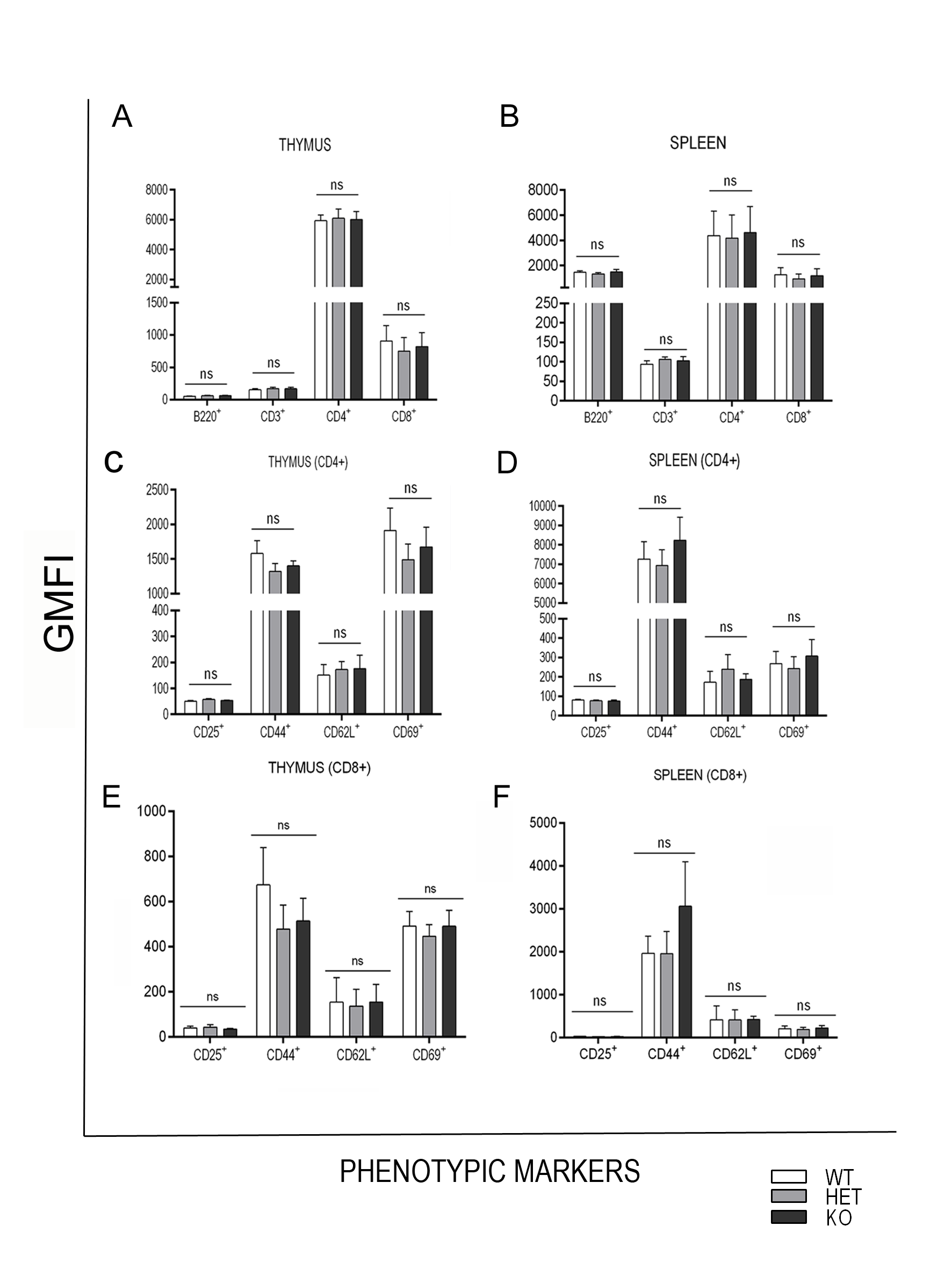

Supplement: S3 Fig — Quantification of B and T cell populations from A) the thymus and B) the spleen using geometric mean fluorescence intensity (GMFI). GMFI quantification of CD4+ T cell activation markers from C) thymocytes and D) splenocytes and CD8+ T cell activation markers from E) thymocytes and F) splenocytes. (TIF) [file pone.0122093.s003.tif]

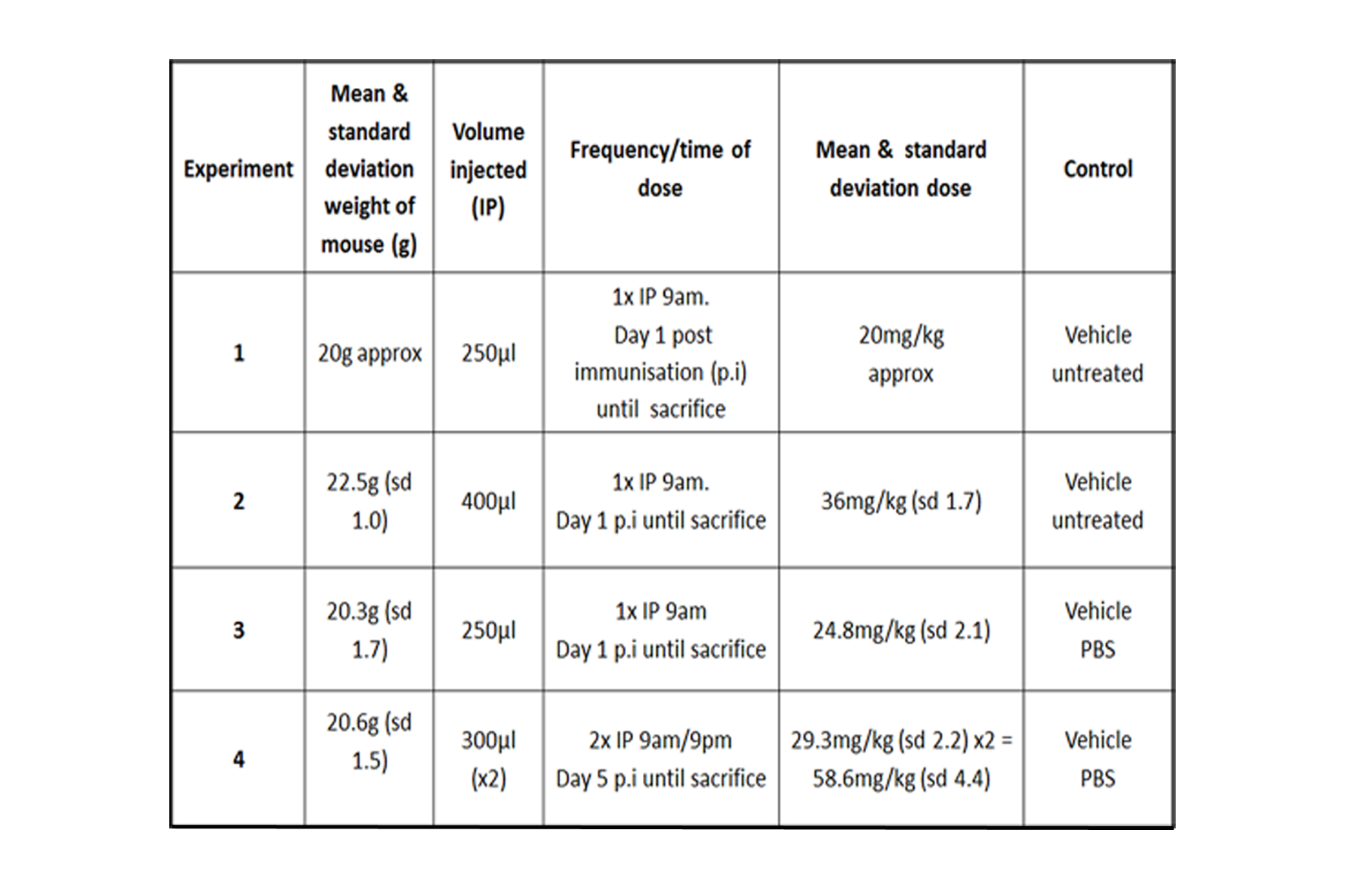

Supplement: S4 Fig — Mice were immunised and randomly allocated to a treatment group. Each mouse was given a standardised daily or twice daily volume of SB-435495 by i.p injection and the dose calculated based on the weight of the animal. (TIF) [file pone.0122093.s004.tif]

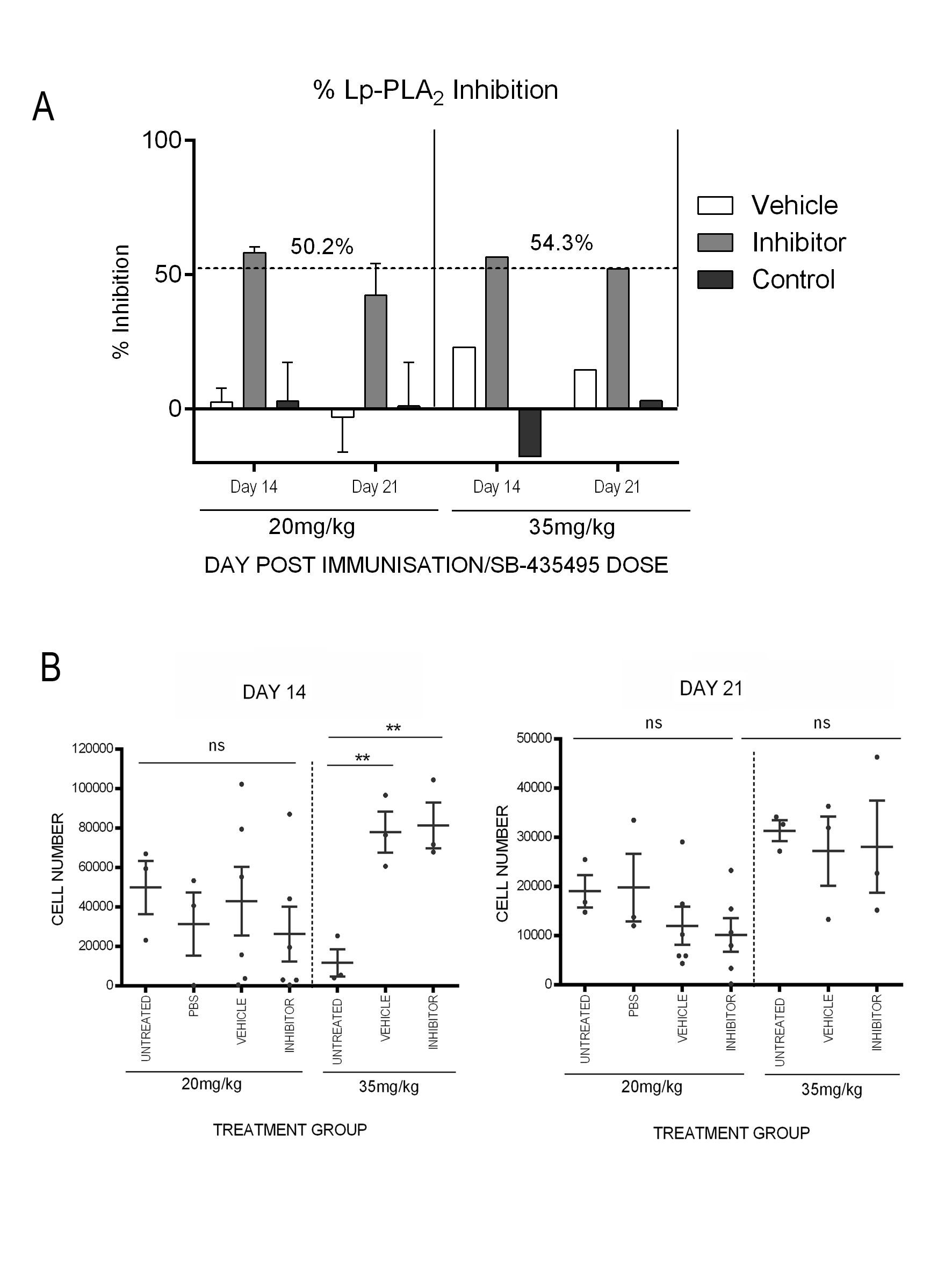

Supplement: S5 Fig — A) Percent inhibition calculated from murine plasma, taken at time of sacrifice (n = 3) B) Quantification of cells infiltrating the retina at day 14 and 21 post immunisation (n = 3) ** p<0.01 (TIF) [file pone.0122093.s005.tif]

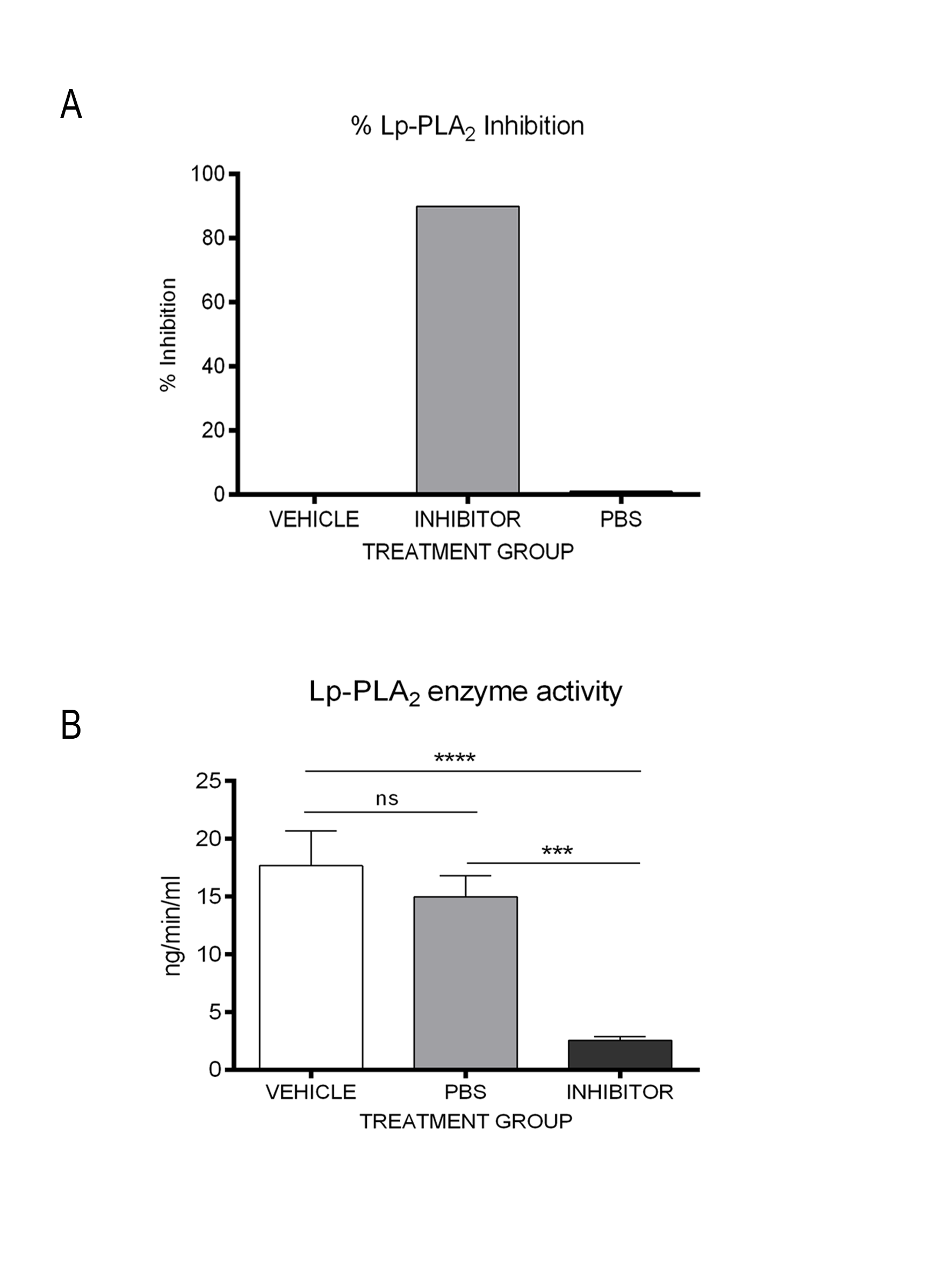

Supplement: S6 Fig — A) % Lp-PLA2 inhibition and B) Lp-PLA2 enzyme activity calculated from murine plasma, taken at time of sacrifice (n = 14–15). *** p<0.005 **** p<0.001 (TIF) [file pone.0122093.s006.tif]
